# Supplementary material for: Cytomolecular Classification of Thyroid Nodules Using Fine-Needle Washes Aspiration Biopsies
Source: Int J Mol Sci. 2022 Apr 9;23(8):4156. doi: 10.3390/ijms23084156 (PMC9028391; doi:10.3390/ijms23084156)
Supplement: Supplementary file 1 [file ijms-23-04156-s001.zip › ijms-1655396-supplementary.pdf]

International Journal of
Molecular Sciences

Article

Cytomolecular Classification of Thyroid Nodules Using Fine-Needle Washes Aspiration Biopsies

Supplementary Figures

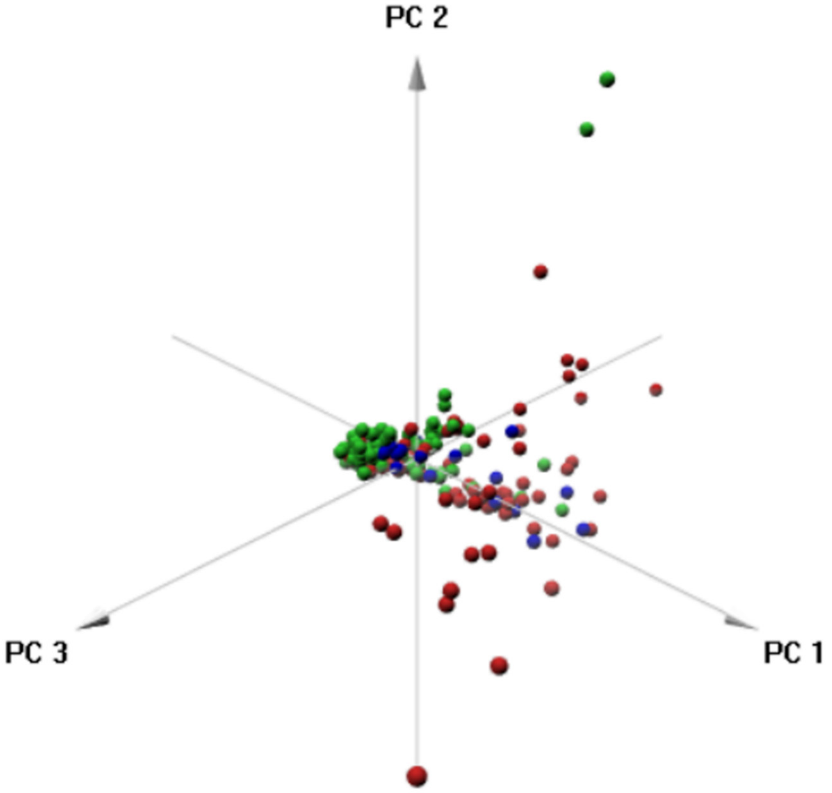

Figure S1. Principal component analysis on 240 thyroid FNA using the overall average spectrum of each sample. Green: benign entities (i.e. HP, HT, B-Goiter), Red: malignant nodules (i.e. PTC, MTC, rare parathyroid tumour), Blue: borderline diagnosis (i.e. NIFTP, FA, WDT-UMP) and Lymph node.

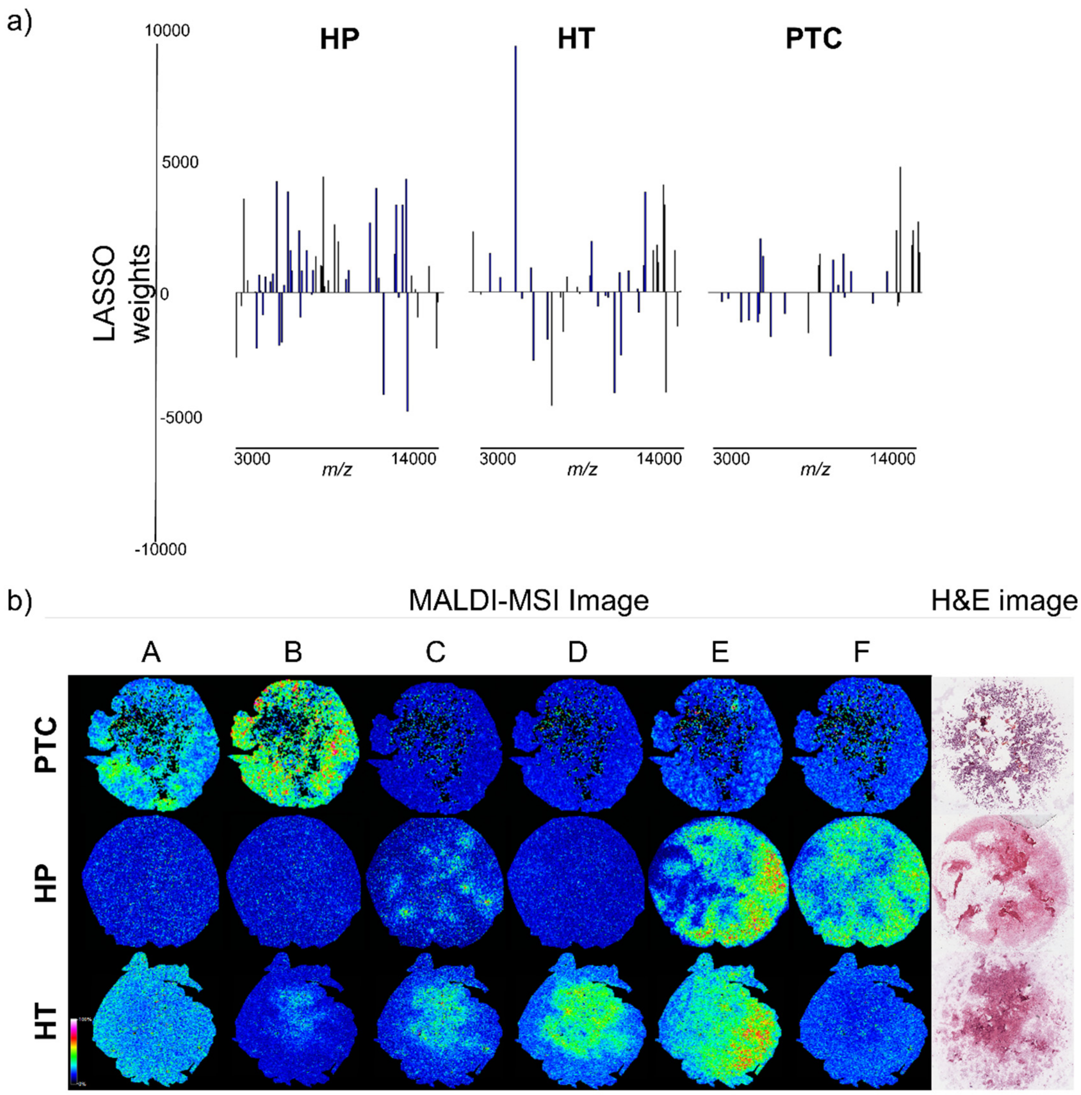

Figure S2. MALDI-MSI analysis of FNA needle washes and statistical analysis. **(a)** 95 m/z features selected by the Lasso model with the corresponding weights in HP, HT and PTC samples. **(b)** HP, HT and PTC MALDI-MSI images of six m/z and their H&E images.

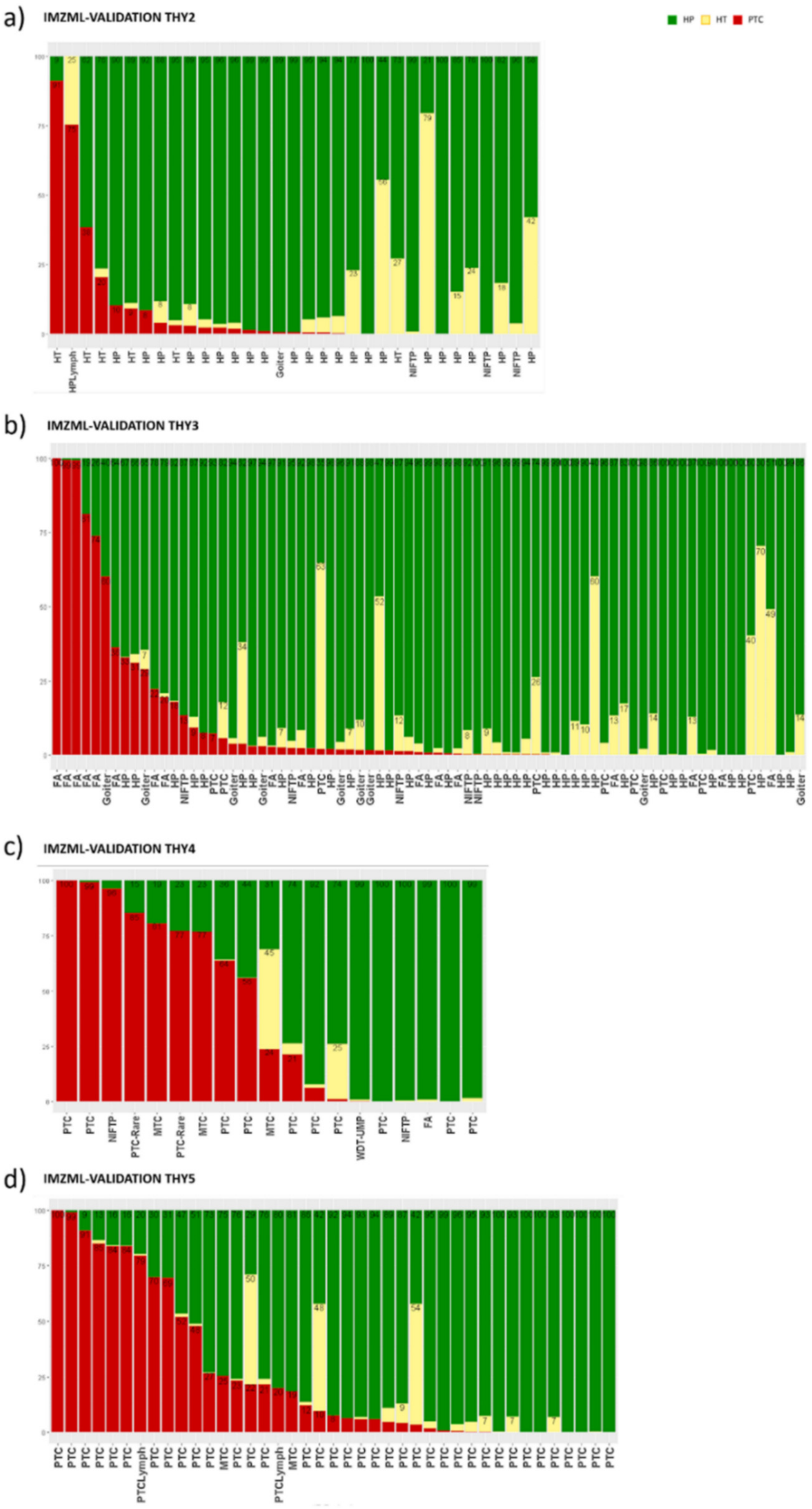

Figure S3. Stacked bar charts of the percentage of pixels in each FNA of the validation set classified based on pixel-by-pixel (IMZML) approach as HP (green), HT (yellow) and PTC (red) in the (a) 33 TIR2/THY2, (b) 77 TIR3/THY3, (c) 19 TIR4/THY4 and (d) 41 TIR5/THY5 samples. Each nodule was classified as malignant when the percentage of red pixels was > 7%.

Supplementary Tables

Table S1. Cytological characteristics of thyroid nodules.

Characteristic	Total (n=240)	Cytology THY2 (n=83)	THY3 (n=77)	THY4 (n=19)	THY5 (n=61)
**Diagnosis**					
**Benign**					
**HP**	96	59	37	0	0
**HP-lymph**	1	1	0	0	0
**B-goiter**	13	4	9	0	0
**HT**	16	16	0	0	0
**FA**	18	0	17	1	0
**NIFTP**	10	3	5	2	0
**WDT-UMP**	1	0	0	1	0
**Malignant**					
**PTC**	76	0	9	10	57
**PTC-lymph**	2	0	0	0	2
**MTC**	5	0	0	3	2
**Rare parathyroid tumour**	2	0	0	2	0
**Nodule Localization**					
**Right Lobe**	122	45	40	12	25
**Left Lobe**	112	35	36	7	34
**Isthmus**	3	2	1	0	0
**Lymph node**	3	1	0	0	2
**Nodule Size (mm)**					
**Mean (SD)**	20.7 (11.3)	21.3 (10.4)	23.7 (13.6)	16.1 (6.8)	17.6 (9.1)
**Range**	5-70	5-60	5-70	8-35	8-50
**Sample cellularity***					
**Poor**	87	18	44	7	18
**Good**	94	40	24	7	23
**Optimal**	59	25	9	5	20

Legend: Goiter, HP-lymph node reported as HP; PTC-lymph and Rare parathyroid tumour, for the purpose of the analysis considered malignant as a PTC.

*Sample cellularity: poor (sample with 20-30% of thyrocytes cells), good (sample with 30-70%) and optimal (sample with >70%).

HP: hyperplastic, HT: Hashimoto thyroiditis, FA: follicular adenoma, PTC: papillary thyroid carcinoma, MTC: medullary thyroid carcinoma, WDT-UMP: well differentiated tumor of uncertain malignant potential, NIFTP: Non-Invasive Follicular Thyroid neoplasm with Papillary-like nuclear features.

Table S2. Predicted diagnosis based on the 605 ROIs of the training set according to histopathology/follow-up.

**Histopathology/ follow-up**	**Prediction**			
		**Benign**		**Malignant**
	**Total**	**HP**	**HT**	**PTC**
**HP**	381	366	5	10
**HT**	50	12	38	0
**PTC**	174	5	2	167

HP: hyperplastic, HT: Hashimoto thyroiditis, PTC: papillary thyroid carcinoma.

Table S3. Performances of the classifier on the three MALDI-MSI approaches in the training set (n=70).

	**TP**	**FP**	**TN**	**FN**	**% Sensitivity (95%CI)**	**% Specificity (95%CI)**	**% PPV (95%CI)**	**% NPV (95%CI)**	**AUC**	**% Accuracy**
**Pixel-by-pixel**	20	2	48	0	100.0 (83.2-100.0)	96.0 (86.3-99.5)	90.9 (70.8-98.9)	100 (92.6-100)	1.00	97.1
**ROIs**	20	0	50	0	100.0 (83.2-100.0)	100 (92.9-100)	100 (83.2-100)	100 (92.9-100)	1.00	100
**Average spectra**	19	0	50	1	95.0 (75.1-99.9)	100 (92.9-100)	100 (82.4-100)	98.0 (89.6-100)	0.98	98.6

Legend: TP=True Positive, FP=False Positive, TN=True Negative, FN=False Negative, PPV=Positive Predictive Value, NPV=Negative Predictive Value, 95%CI=95% Confidence Interval, AUC=Area Under the Curve

Table S4. Predicted diagnosis based on the 3 levels classification strategy in the pixel-by-pixel analysis according to histopathology/follow-up for a) the training set and b-e) the validation set by the Bethesda reporting system.

**a) Training (n=70)**	**Prediction**			
**Histology/FUP**	**Total**	**Benign**	**Gray Zone**	**Malignant**
**Benign**				
**HP**	40	35*	3	2
**HT**	10	10	0	0
**Malignant**				
**PTC**	20	0	0	20

*3 Goiter.

**b) TIR2 (n=33)**	**Prediction**			
**Histology/FUP**	**Total**	**Benign**	**Gray Zone**	**Malignant**
**Benign**				
**HP**	24	21*	2	1#
**HT**	6	2	1	3
**FA**	0	0	0	0
**NIFTP**	3	3	0	0
**WDT-UMP**	0	0	0	0

*1 Goiter, #1 HP-lymph node.

**c) TIR3 (n=77)**	**Prediction**			
**Histology/FUP**	**Total**	**Benign**	**Gray Zone**	**Malignant**
**Benign**				
**HP**	46	39*	2	5#
**HT**	0	0	0	0
**FA**	17	9	0	8
**NIFTP**	5	4	1	0
**WDT-UMP**	0	0	0	0
**Malignant**				
**PTC**	9	8	1	0
**MTC**	0	0	0	0

*7 Goiter, #2 Goiter.

**d) TIR4 (n=19)**	**Prediction**			
**Histology/FUP**	**Total**	**Benign**	**Gray Zone**	**Malignant**
**Benign**				
**HP**	0	0	0	0
**HT**	0	0	0	0
**FA**	1	1	0	0
**NIFTP**	2	1	0	1
**WDT-UMP**	1	1	0	0
**Malignant**				
**PTC**	12	5	0	7°
**MTC**	3	0	0	3

°2 rare parathyroid tumour.

**e) TIR5 (n=41)**	**Prediction**			
**Histology/FUP**	**Total**	**Benign**	**Gray Zone**	**Malignant**
**Malignant**				
**PTC**	39	20	3	16#
**MTC**	2	0	0	2

#2 PTC-lymph node.

HP: hyperplastic, HT: Hashimoto thyroiditis, FA: follicular adenoma, PTC: papillary thyroid carcinoma, MTC: medullary thyroid carcinoma, WDT-UMP: well differentiated tumor of uncertain malignant potential, NIFTP: Non-Invasive Follicular Thyroid neoplasm with Papillary-like nuclear features.
